# Functional Analysis and Molecular Dynamics Simulation of LOX-1 K167N Polymorphism Reveal Alteration of Receptor Activity

**DOI:** 10.1371/journal.pone.0004648

**Published:** 2009-02-27

**Authors:** Silvia Biocca, Mattia Falconi, Ilaria Filesi, Francesco Baldini, Lucia Vecchione, Ruggiero Mango, Francesco Romeo, Giorgio Federici, Alessandro Desideri, Giuseppe Novelli

**Affiliations:** 1 Department of Neuroscience, University of Tor Vergata, Rome, Italy; 2 Department of Biology, University of Tor Vergata, Rome, Italy; 3 Center of Biostatistics and Bioinformatics, University of Tor Vergata, Rome, Italy; 4 Department of Biopathology and Diagnostic Imaging and Centre of Excellence for Genomic Risk Assessment in Multifactorial and Complex Diseases, School of Medicine, University of Tor Vergata, Rome, Italy; 5 Department of Internal Medicine, University of Tor Vergata, Rome, Italy; 6 IRCCS Humanitas, Rozzano, Milano; 7 Department of Internal Medicine, University of Arkansas for Medical Sciences and Central Arkansas Veterans Healthcare System, Little Rock, Arkansas, United States of America; University of Giessen Lung Center, Germany

## Abstract

The human lectin-like oxidized low density lipoprotein receptor 1 LOX-1, encoded by the *ORL1* gene, is the major scavenger receptor for oxidized low density lipoprotein in endothelial cells. Here we report on the functional effects of a coding SNP, c.501G>C, which produces a single amino acid change (K>N at codon 167). Our study was aimed at elucidating whether the c.501G>C polymorphism changes the binding affinity of LOX-1 receptor altering its function. The presence of p.K167N mutation reduces ox-LDL binding and uptake. Ox-LDL activated extracellular signal-regulated kinases 1 and 2 (ERK 1/2) is inhibited. Furthermore, ox-LDL induced biosynthesis of LOX-1 receptors is dependent on the p.K167N variation. In human macrophages, derived from c.501G>C heterozygous individuals, the ox-LDL induced LOX-1 46 kDa band is markedly lower than in induced macrophages derived from c.501G>C controls. Investigation of p.K167N mutation through molecular dynamics simulation and electrostatic analysis suggests that the ox-LDL binding may be attributed to the coupling between the electrostatic potential distribution and the asymmetric flexibility of the basic spine residues. The N/N-LOX-1 mutant has either interrupted electrostatic potential and asymmetric fluctuations of the basic spine arginines.

## Introduction

Many biochemical and functional studies have suggested a fundamental role of oxidized low density lipoproteins (ox-LDL) and of their main receptor LOX-1 (oxidized low density lipoproteins receptor 1) in the pathogenesis of atherosclerosis [1], [2].

LOX-1 is a disulfide-linked homodimeric type II transmembrane receptor belonging to the C-type lectin family of scavenger receptors. Each subunit is composed by a short 34-residue cytoplasmic region, a single transmembrane segment, and an extracellular 80-residue “neck” domain, predicted to have a coiled coil structure, followed by a 130-residue C-terminal C-type lectin-like domain (CTLD) [3]. The two CTLD domains form a heart-shaped homodimer, consisting of two antiparallel β-sheets flanked by two α-helices with three large loops protruding into the solvent. This fold is stabilized by three conserved intra-chain disulfide bonds and an inter-chain disulfide bridge, located at the N-terminus [4], [5]. On the basis of this structure LOX-1 has been hypothesized to interact with ox-LDL with a 3∶1 stoichiometry [4].

It is expressed in endothelial cells, smooth muscular cells, monocytes/macrophages, platelets, fibroblasts and cardiomyocites [3], [6]–[8]. LOX-1 activation elicits endothelial dysfunction, a key step in the initiation of atherosclerosis, favouring generation of reactive oxygen species, inhibition of nitric oxide synthesis, and enhancement of monocyte adhesion to activated endothelial cells [9]. In addition, LOX-1 is involved in foam cells formation and in inducing smooth muscle cell migration, proliferation and transformation [1].

In vascular endothelial cells, upon recognition of ox-LDL, LOX-1 stimulates several intracellular signaling pathways, including protein kinases such as p38 (MAPK), protein kinase C and extracellular-signal-regulated kinase (ERK) 1/2 [10]–[13]. These signaling pathways activate transcription factor NF-kB, which elicits pro-inflammatory and pro-apoptotic gene expression [14] contributing to the altered cellular function associated with atherogenesis and plaque vulnerability. Recently, several association studies have characterized various polymorphisms (SNPs, single nucleotide polymorphisms) in *OLR1* gene, that encodes for LOX-1 receptor [15]–[17]. It was shown that a linkage disequilibrium block of SNPs located in the *OLR1* gene introns 4, 5, and the 3′ untranslated region are associated to an increased susceptibility to acute myocardial infarction (AMI). These SNPs modulate the expression of a splicing isoform of LOX-1 receptor, named LOXIN, which protects macrophages against ox-LDL-mediated apoptosis [17]. LOXIN is deficient in ox-LDL binding activity but interacts with LOX-1 receptors inhibiting its function through the formation of non-functional hetero-oligomers [18]. However, conflicting results have been reported on the association between some polymorphisms in *OLR1* gene and coronary artery disease (CAD)/AMI susceptibility on the basis of study design, statistical analysis and interpretation of results [19]. In particular, a predicted functional SNP, the G>C transition at position 501 in the exon 4 has been studied, with different conclusions, as a possible valid genomic biomarker for potential CAD/AMI risk factor [15], [20]–[23]. This SNP results in the Lys to Asn amino acid residue replacement at position 167 of the C-type lectin-like domain, in the extracellular portion of LOX-1 receptor. Since this is the ligand binding domain, the p.K167N variation may affect LOX-1 receptor response.

In order to test the effects of the p.K167N SNP, we investigated, at a molecular level, whether the c.501G>C polymorphism could affect LOX-1 receptor activity. Here we report the heterologous expression and functional characterization of wild–type (K/K167) and mutated (N/N167 and K/N167) LOX-1 in different cell lines, including fibroblasts and human endothelial cells. K167N mutation of LOX-1 receptor markedly alters ox-LDL binding, uptake and its intracellular signalling. Investigation of p.K167N mutation through molecular dynamics (MD) simulations, coupled with a time evolution analysis of electrostatic potential, provides an explanation for the drastic reduction of LOX-1 function.

## Results

### Expression of N167-V5 tagged variant

**Figure 1:**
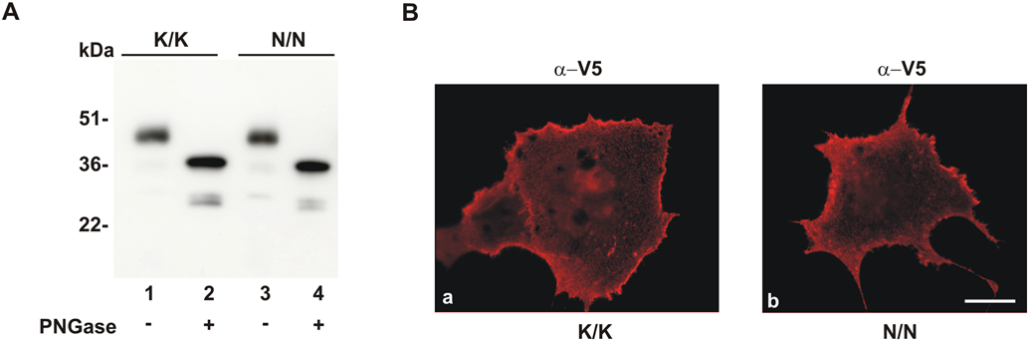
Ectopic expression of K/K167 and N/N167 LOX-1 variants. (A) Western blot analysis of lysates from COS cells transiently transfected with wt-LOX-1-V5 (lanes 1–2) and N/N167 LOX-1-V5 (lanes 3–4) incubated with PNGase as indicated and immunoblotted with Mab anti-V5. Molecular weight markers are given in kilodaltons (kDa) and are indicated on the left. (B) Surface localization of wt-LOX-1-V5 (panel a) and N/N167 LOX-1-V5 (panel b) expressed in COS cells was analyzed by indirect immunofluorescence with Mab anti-V5. Scale bar 10 µm.

The native K/K167 (wt-LOX-1-V5) and mutated N/N167 (mut-LOX-1-V5) proteins have been ectopically expressed in different cell lines, including fibroblasts (COS) and human endothelial cells (HUVEC) and studied by Western blot. As shown in Figure 1A, an intense band at 48 kDa is visualized with anti-V5 antibodies in extracts derived from COS cells expressing wt-LOX-1-V5 (lane 1) and mut-LOX-1-V5 (lane 3). In some gels the 48 kDa protein is resolved as a doublet at 48 and 46 kDa and an additive product is observed at 37 kDa (weak band in lanes 1 and 3). This is a degradation product immunoreactive with anti-LOX-1 polyclonal antibodies. Removal of N-linked glycans by PNGase treatment results in a band at 36 kDa and at 25 kDa, representing the full length deglycosylated receptor and a smaller degraded product of LOX-1 respectively (Figure 1A, lane 2). An identical pattern is observed for mut-LOX-1-V5 (lane 4).

The two isoforms have been labelled for surface receptors in live cells 24 hours after transfection (Figure 1B). A similar punctuate plasma membrane-associated fluorescence is seen in cells expressing K/K LOX-1 (panel a) and N/N mut-LOX-1 (panel b). As expected, when we compared the intracellular distribution of wt-LOX-1 and mut-LOX-1 in fixed and permeabilized transfected cells, we observed the same intracellular localization (not shown). It is worth noting that similar patterns of expression were also obtained in human endothelial cells HUVEC (data not shown).

Altogether these results indicate that the native and mutated LOX-1 receptor undertake the same glycosylation/maturation process and trafficking pathway.

### Wt-LOX-1 and N167 variant interact and form hetero-oligomers *in vivo*

**Figure 2:**
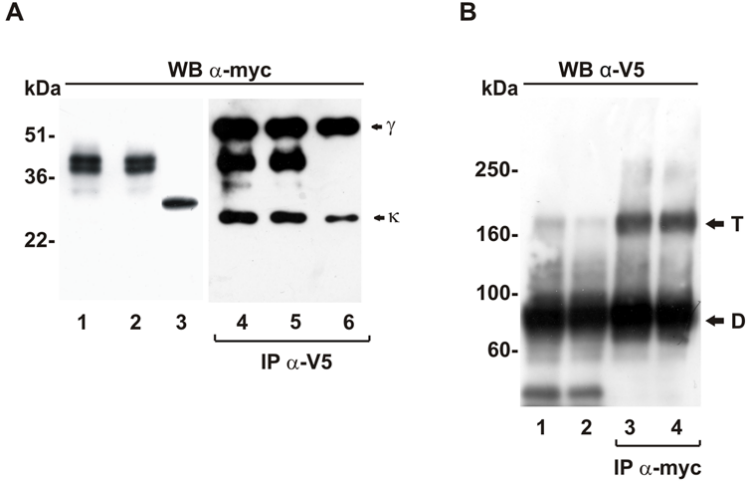
In vivo interaction between wt-LOX-1 and mutated LOX-1 receptors. (A) COS cells were co-transfected with wt-LOX-1-myc and wt-LOX-1-V5 (lanes 1 and 4), N/N167 LOX-1-V5 and wt-LOX-1-myc (lanes 2 and 5) and Sec-8H4-myc and wt-LOX-1-V5 (lanes 3 and 6) at a DNA ratio 1∶1. Half lysates were immunoblotted with anti-myc antibodies (lanes 1–3) and the remaining extracted proteins were first immunopurified with anti-V5 antibodies and then probed with anti-myc 9E10 (lanes 4–6). The migration of molecular weight markers (kDa) is indicated on the left and the heavy (γ) and the light (κ) chains are indicated on the right. (B) Lysates of COS cells co-expressing wt-LOX-1-myc and wt-LOX-1-V5 (lane 1 and 3) and wt-LOX-1-myc and N/N167 LOX-1-V5 (lanes 2 and 4) were separated by a NuPAGE 3–8% polyacrylamide gradient gel in non reducing conditions. Lanes 1 and 2 represent the extracts immunoblotted with Mab anti-V5, while lanes 3 and 4 are the extracted proteins first immunopurified with Rabbit anti-myc and then probed with Mab anti-V5. The dimers and tetramers are indicated, respectively, with D and T and the migration of molecular weight markers (kDa) is indicated on the left. All experiments were repeated three times with similar results.

The *in vivo* interaction between wt-LOX-1 and mut-LOX-1 isoforms in transfected cells has been studied by co-immunoprecipitation analysis. Constructs expressing myc-tagged wt LOX-1, the non-relevant Sec-8H4-myc and the V5-tagged N/N167 proteins have been transiently co-transfected in COS cells, that were processed for analysis 24 hours after transfection. Cellular lysates were first immunoprecipitated with anti-V5 antibodies, separated by SDS-PAGE and then immunoblotted with anti-Myc Mab 9E10 to reveal wt-LOX-1-myc and Sec-8H4-myc. The transfection efficiency and specificity of the experiment were confirmed by analysing the input of LOX-1-myc and Sec8H4-myc in extracts before immunoprecipitation. As it can be seen in Figure 2A, a similar amount of myc-tagged proteins is present in all samples (lanes 1–3). After immunoprecipitation with anti-V5 antibodies, the 48 and 46 kDa bands, corresponding to LOX-1-myc, are observed either in extracts derived from cells expressing LOX-1-V5 and in cells expressing mut-LOX-1-V5 (Figure 2B, lanes 4 and 5). On the contrary, the non-relevant Sec8H4-myc protein, used as a control, does not co-immunoprecipitate with LOX-1-V5 (lane 6).

To detect and separate high molecular weight forms of LOX-1, 3–8% acrylamide gels have been used in non reducing conditions. As it can be seen in Figure 2B (lane 1), in COS cells co-expressing myc- and V5- tagged K/K167 LOX-1, the wild type receptor is expressed as a major 90 kDa band corresponding to the homo-dimer and a minor band at 180 kDa corresponding to the homo-tetramer. The expression of N/N167-LOX-1-V5 in the presence of K/K-LOX-1-myc at a ratio 1∶1 (lane 2) or expression of N/N167 mutated LOX-1 alone (not shown) results in a similar pattern, indicating the formation of higher molecular weight (MW) oligomers.

In order to assess whether the two receptor isoforms form stable hetero-oligomers, lysates derived from COS cells co-expressing myc- and V5-tagged wt-LOX-1 and mut-LOX-1 were first co-immunoprecipitated with anti-myc antibodies and then analysed by Western blot with anti-V5 antibodies. As shown in Figure 2B, one major band at 90 kDa corresponding to LOX-1 homo-dimers and a 180 kDa band corresponding to the homo-tetramers are present when wt-LOX-1-myc and wt-LOX-1-V5 were co-transfected (lane 3), as also previously reported [18]. Immunoprecipitation of lysates derived from COS cells co-expressing wt-LOX-1-myc and N/N167-LOX-1-V5 at a ratio 1∶1 results in the co-immunoprocipitation of the 90 kDa and 180 kDa bands, corresponding to the wt-LOX-1/mut-LOX-1 hetero-dimers and hetero-tetramers (lane 4).

### Ox-LDL binding and uptake in wt-LOX-1 and mut-LOX-1 expressing cells

**Figure 3:**
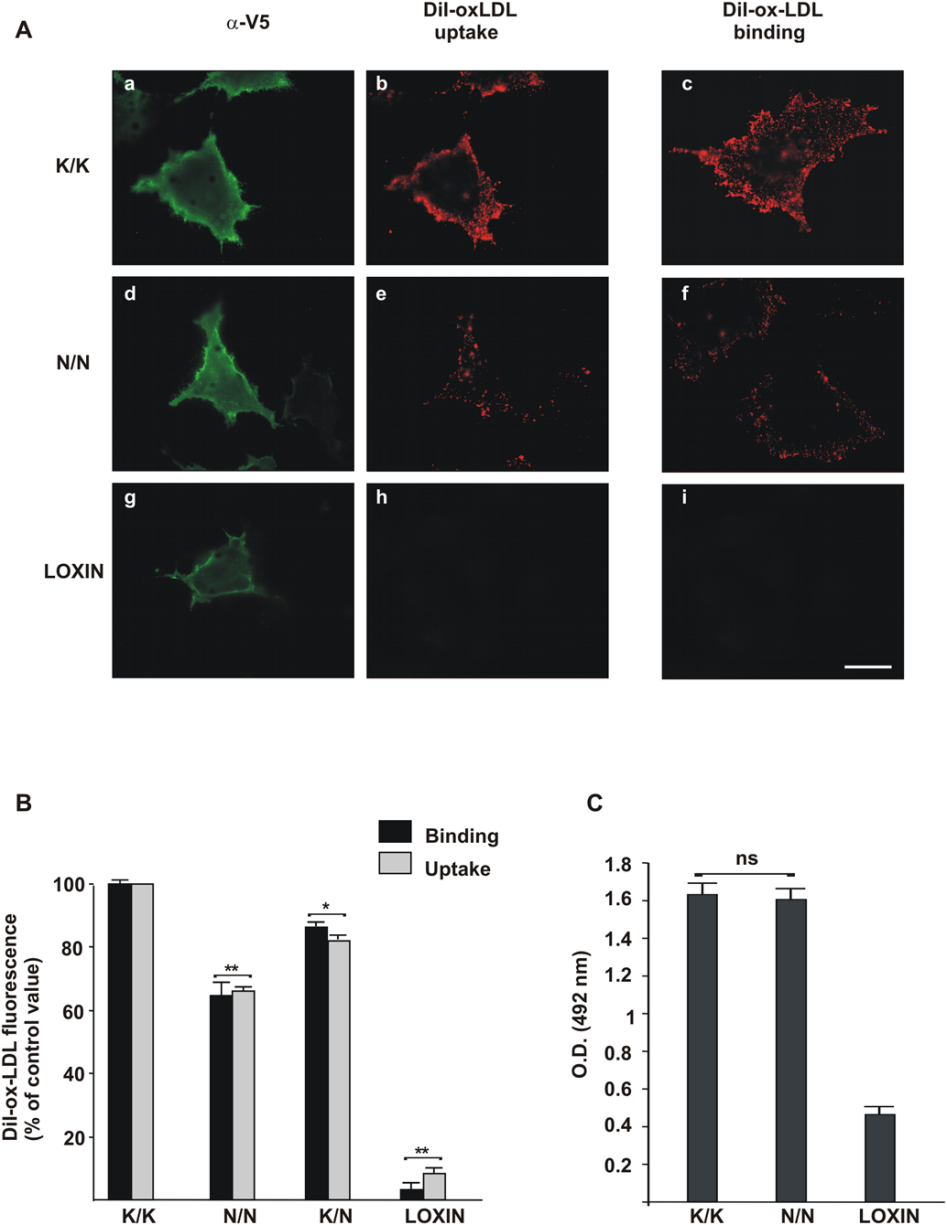
Effect of K167N mutation on LOX-1 ox-LDL binding and uptake. (A) Cells were incubated with 10 µg/ml of DiI-ox-LDL for 4 h at 37°C (uptake) or 1 h on ice (binding). Surface expressed K/K167-V5, N/N167-V5 or LOXIN-V5 proteins were visualized with Mab anti-V5 (panels a, d and g respectively). Fluorescence of DiI-ox-LDL is shown in panels b, e and h in the uptake assay and in panels c, f and i in the binding assay. A strong and specific fluorescence of DiI-ox-LDL was detected in cells transfected with K/K167 LOX-1 (panels b and c), while a lower signal was detected in cells expressing N/N167 (panels e and f). No fluorescence was detected in cells expressing LOXIN (panels h and i). Scale bar 10 µm. (B) Binding and uptake of DiI-ox-LDL in cells expressing K/K167, N/N167, K/N167 or LOXIN were measured by extraction of DiI fluorescence with isopropanol as described in Materials and methods. (C) Surface receptors were measured by cytoELISA assay by using anti-V5 antibodies, as described in the text. The data represent the average±standard deviation (SD) of four separate experiments. A p value (** p<0.01; * p<0.05) was considered to be statistically significant; the ns label indicates no significant difference.

COS cells transfected with wt-LOX-1, mut-LOX-1 or LOXIN have been incubated with DiI-ox-LDL at 4°C for 1 hour or at 37°C for 4 hours, to investigate the ox-LDL binding and uptake respectively. DiI-ox-LDL efficiently binds to wild type LOX-1 receptors (Figure 3A, panel c). Incubation of these cells for 4 hours at 37°C with DiI-ox-LDL leads to its uptake and accumulation inside cells (panel b). Interestingly, a lower efficiency of DiI-ox-LDL binding and uptake is observed in cells expressing the mut-LOX-1 variant (panels e and f). No fluorescence can be visualized in panels h and i, confirming that LOXIN expressing cells do not bind DiI-ox-LDL [18].

Quantification of bound DiI-ox-LDL has been obtained by its extraction with isopropanol and spectrofluorometric analysis. As shown in Figure 3B, the decrease of ox-LDL binding and uptake is 15–20% in cells transfected with K/K167 and N/N 167-LOX-1 isoforms at a ratio 1∶1 and 30–35% in cells expressing the N/N167 variant alone, when compared to the native protein. As previously reported [18], the inhibition of ligand binding and its uptake is almost complete in LOXIN expressing cells (Figure 3B). Notably, as it occurs in cells that were simultaneously transfected with LOX-1 and LOXIN, the co-transfection of K167N and LOXIN at a ratio 1∶1 leads to a marked inhibition of the receptor binding activity (18 and data not shown).

We have also quantified the surface appearance of wt-LOX-1, the two mut-LOX-1 variants and LOXIN by a cytoELISA assay. The missense K167N mutation does not give any effect on LOX-1 receptors trafficking to the plasma membrane and a similar signal is observed in cells transfected with wt-LOX-1 and mut-LOX-1 (Figure 3C). It is worth noting that, although the K167N variant has a lower ox-LDL receptor activity, the amount of exposed receptors is identical to the wild type LOX-1 receptor. On the contrary, only 25% LOXIN molecules are exposed, confirming that the LOXIN splice variant localizes mostly intracellularly [18].

### ERK 1/2 activity is impaired by single K167N mutation

**Figure 4:**
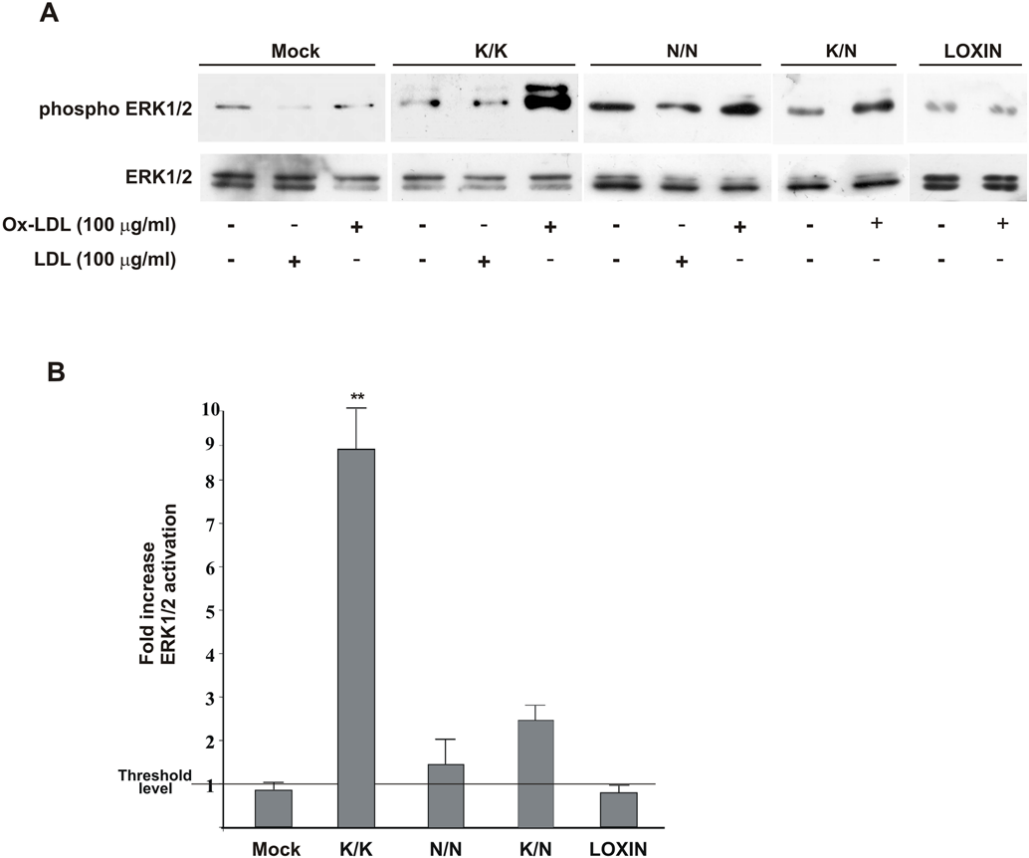
Ox-LDL stimulated ERK 1/2 phosphorylation in COS cells. (A) COS cells were transiently transfected with pEF/V5-His empty vector (Mock) or K/K167, N/N167, K/N167 or LOXIN variants. 48-h after transfection, cells were incubated with 100 µg/ml of LDL or ox-LDL, as indicated, for 10 minutes at 37°C. Cell extracts were immunoblotted with mouse anti-phospho-ERK 1/2 and rabbit anti ERK1/2.(B) Densitometric measurements were performed to evaluate the fold increase in phospho-ERK 1/2 activation in the presence of ox-LDL. The data represent the average±standard deviation (SD). A p value (** p<0.01) was considered to be statistically significant.

To evaluate whether single K167N mutation on LOX-1 receptor impairs ERK 1/2 kinase activity, we analyzed ERK 1/2 phosphorylation by Western blot with specific antibodies directed against the phosphorylated ERK 1/2 [12], [13]. Wild-type K/K-LOX-1, mutated N/N-LOX-1 and K/N-LOX-1 or LOXIN have been transiently transfected in COS cells, incubated with or without LDL or ox-LDL at different concentrations (from 10 to 100 µg/ml) and the phosphorylated-ERK 1/2 bands compared. Incubation with 100 µg/ml of ox-LDL for 10 min gives the optimal conditions for maximal LOX-1 dependent ERK 1/2 activation without cytotoxicity (not shown). Figure 4A shows the result of one typical experiment. The fold increase in ERK phosphorylation was evaluated by densitometric analysis of the 42 kDa phospho-ERK band (Figure 4B). As it can be seen, ox-LDL stimulates ERK phosphorylation through wt-LOX-1 receptors and the fold increase of activation is 8–10, when compared to the intensity of the band observed in lysates derived from non treated cells or cells treated with 100 µg/ml of LDL for 10 min. This value is 1,2–2 in N/N-LOX-1 and 2–3 in K/N-LOX-1 expressing cells, indicating a marked decrease in the activation of ERK pathway. Incubation with LDL did not induce phosphorylation of ERK 1/2 kinases in cells transfected with K/N167 and LOXIN (not shown). It is worth noting that, as expected, mock transfected cells and cells expressing LOXIN variant do not show any induction of phosphorylation of ERK 1/2 kinases.

### Ox-LDL dependent LOX-1 induction is altered in macrophages derived by c.501G>C heterozygous individuals

**Figure 5:**
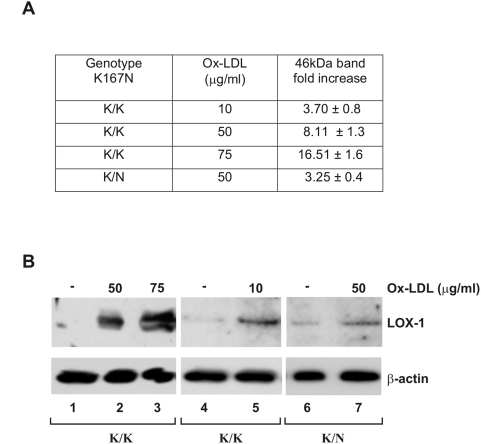
Ox-LDL-dependent LOX-1 induction in human macrophages derived by G501G and G501C individuals. (A) Densitometric measurements (mean±SD) were performed to evaluate the fold increase in LOX-1 46 kDa band intensity. The endogenous expression of LOX-1 was analysed by Western blotting with rat anti-human LOX-1 antiserum in human macrophages derived from 10 K/K and 3 K/N individuals incubated in the absence or in the presence of 10, 50 or 75 µg/ml ox-LDL, as indicated. (B) Western blot of macrophages derived from K/K control and K/N heterozygous individuals. Protein level was monitored by probing the same blot with anti-β-actin IgG. In lane 3 β-actin may resemble a doublet for a small rupture of the gel.

Human monocytes have been isolated from peripheral blood mononuclear cells (PBMCs) from buffy coats of volunteers, genotyped for the presence of K167N SNP and cultured *in vitro* for 11 days in order to promote their transition to macrophages, before ox-LDL induction. Ox-LDL-dependent induction of endogenous LOX-1 receptors was studied by Western blot with anti-LOX-1 polyclonal antiserum (Figure 5B). The intensity of the 46 kDa LOX-1 band in lysates derived from ox-LDL-treated cells has been measured by densitometric analysis and compared to the band present in lysates derived from non treated cells. As it can be seen, LOX-1 band is induced in human differentiated macrophages derived from wild type volunteers (c.501G) in an ox-LDL dose-dependent manner. Incubation with 10 µg/ml of ox-LDL results in 3–4,5 fold increase in band intensity, 50 µg/ml in 6,8–9,4 and 75 µg/ml in 15–18 fold increase (Figure 5A). Interestingly, induction of LOX-1 receptor band was also dependent on the p.K167N SNP genotype. When differentiated macrophages derived from c.501G>C (KN) patients have been cultured in the absence or in the presence of 50 µg/ml ox-LDL and analyzed for LOX-1 induction, the 46 kDa LOX-1 band increases of 2,8–3,6 fold (Figure 5A). This value is much lower than that obtained in wild type c.501G (KK) derived macrophages and is similar to the intensity obtained from these macrophages induced with 10 µg/ml of ox-LDL (Figure 5B, compare lanes 5 and 7).

### Positive electrostatic potential and asymmetric fluctuations of basic spine arginines are altered in the K167N mutants

In order to identify the structural-dynamical properties that are responsible for altered receptor activity, comparative MD simulations of the K/K, N/N and K/N-LOX-1 CTLD domain have been carried out.

The proteins have been analyzed in the last 10 ns of the trajectory (i.e. from 5 ns to 15 ns), where the three systems reach a conformational stability, as monitored by time evolution of the global Root Mean Square Deviation (RMSD), i.e. the displacement from the starting structure (see Figure S1 of Supplementary Data).

Mutation of K167 to N leaves the charged residue E170 uncoupled. In fact the salt bridge between K167 and E170, present in the X-ray structure and stable in the wild type simulation, is removed in the N/N-LOX-1 simulation and is present only in the wild type monomer of the K/N-LOX-1 simulation. The residue E170, once lost its partner K167, is unable to form a new stable salt bridge with any other positively charged residue. E170, as a consequence of mutation, remains as a free negative charge fluctuating on the LOX-1 surface, influencing the electrostatic potential generated by the arginines of the basic spine.

**Figure 6:**
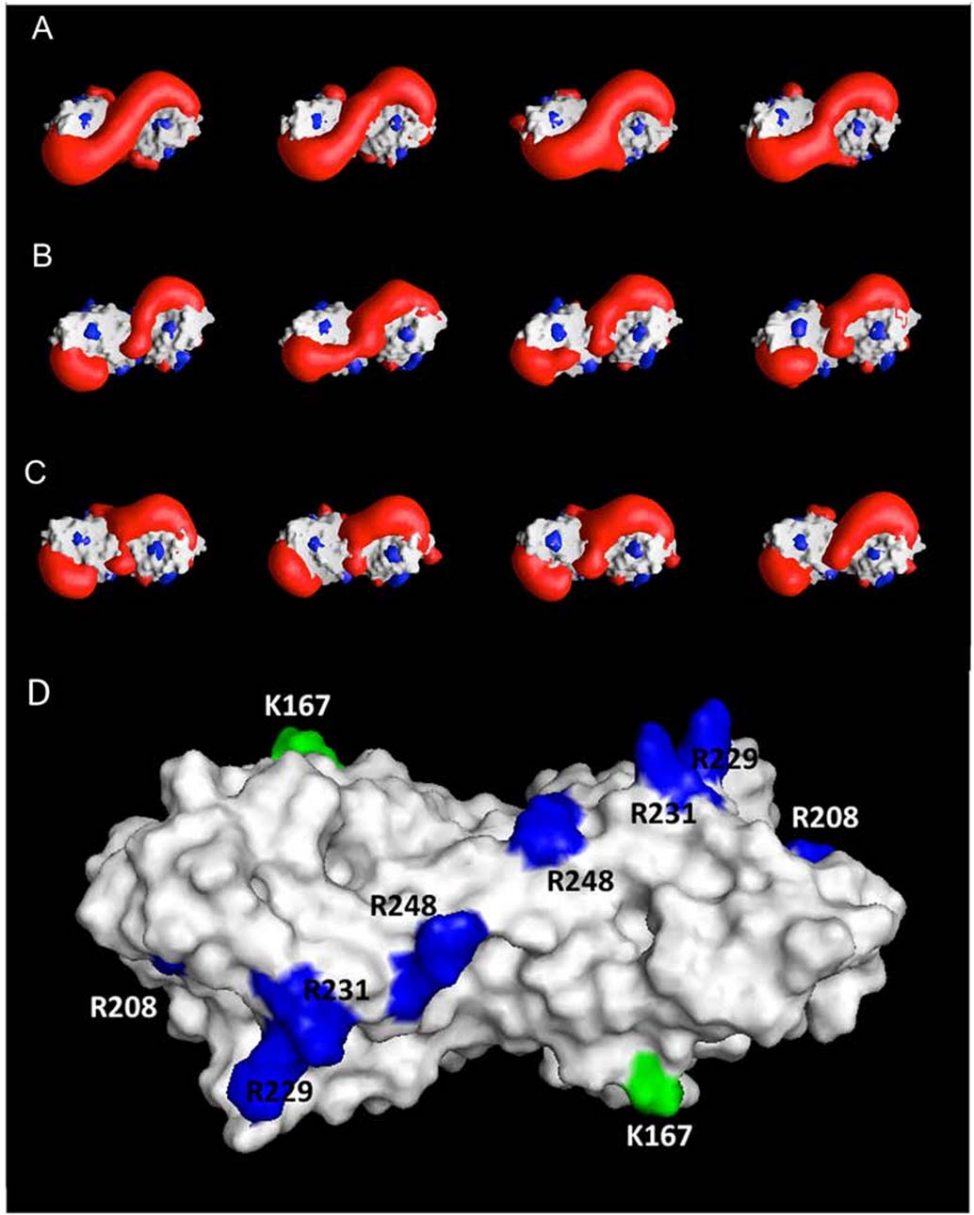
GRASP representations of the isopotential electrostatic distribution observed along the trajectory around (A) K/K167, (B) N/N167 and (C) K/N167 LOX-1 mutants. Red surfaces represent the positive isopotential surface at 2.0 kT/e (where k is the Boltzmann constant, T = 298 K and 1kT/e = 0.0257 V), blue surface the corresponding negative values. The protein boundaries are represented by a rendered molecular surface. Isopotential surfaces in the protein interior are not drawn for clarity. Panel D shows the molecular surface of the CTLD domain representing the location of the arginines composing the basic spine (blue) and the mutation site (green).

The electrostatic potential contour diagram of the CTLD domains has been calculated at 2.0 kT/e for 20 representative configurations each extracted every 500 ps from the trajectories. Figure 6 shows the potentials for 4 representative configurations (i.e. snapshots at 1.5, 4.0, 6.5 and 9.0 ns). The potential surface for the wt-LOX-1 protein is wide and characterized by positive values above the basic spine region (red isopotential surface in Figure 6A and blue arginine residues in panel D), in line with the proposed electrostatic recognition with the negatively charged surface of ox-LDL [4], [5]. On the contrary, K167 mutants show alteration on the potential surface over the ox-LDL recognition site, i.e. over the basic spine arginines that are distant from 23 to 51 Å from the mutation site (Figure 6D). In the N/N and K/N-LOX-1 variants (Figure 6B and 6C, respectively) the potential is less regular when compared to the wild type. It has a reduced positive lobes volume and a larger variability when observed as a function of time. This result well correlates with the lower ox-LDL recognition displayed by the N/N and the K/N mutants described in Figure 3.

**Figure 7:**
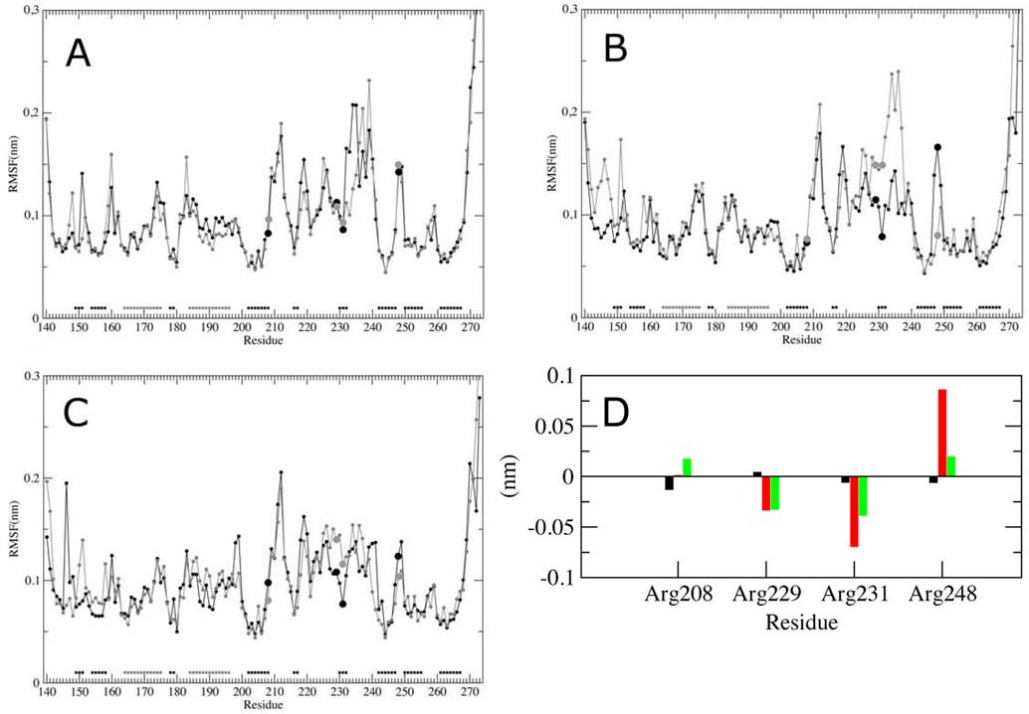
Average main chain residue RMSF of the two subunits of the CTLD domain of (A) K/K167, (B) N/N167 and (C) K/N167 LOX-1. The A subunit is shown by a black line with black filled circles, while the B subunit is indicated by a grey line with gray filled circles. In panel C the black and the grey lines represent the mutated and the wild type subunit, respectively. The arginines belonging to the basic spine are indicated by large filled circles. The residues that in the X-ray starting structure are in α-helix and β-strand are indicated by the grey and black squares close to the X-axis, respectively. (D) Differences between the average RMSF values of the basic spine arginine residues belonging to the two subunits of the CTLD domain: wt (black), N/N (red) and K/N LOX-1 (green). The first subunit value is subtracted to the second subunit value.

The fluctuations of the residues (i.e. the standard deviation of residue position during the simulation) have been also monitored from the MD simulations focusing the attention on the basic spine residues. The main chain root mean square fluctuations (RMSFs), calculated over the trajectories and averaged over each residue, for the wt-LOX-1 and the two N/N and K/N-LOX-1 mutants, indicate that most residues have fluctuations not higher than 0.2 nm, apart the C-terminal tails which reach values around 0.35 nm (Figure 7 A,B,C). The N-terminal tails are less flexible due to the presence of the disulfide bridge between Cys140 of the subunits A and B and reach values lower than 0.2 nm. In the three proteins a relatively highly fluctuating region (values between 0.14 and 0.25 nm) is localized between Arg209 and Gly241, including the loops L1, L2 and L3 and the two small β-strands: β2a and β2b [4], [5]. Although the three proteins display a similar pattern of mobility, differences in fluctuations between the wt-LOX-1 and the mutants are observed for the residues belonging to the basic spine (i.e. arginines 208, 229, 231, 248). In the two subunits of the wild type (Figure 7, panels A and D) the average fluctuation of the arginines pairs is almost identical (the absolute value of their difference ranging from 0.0 to 0.01 nm). On the contrary, in the two monomers of the N/N-LOX-1 mutant (Figure 7, panels B and D) the arginines pairs fluctuate differently. In detail, Arg229, 231 and 248 show an absolute value of their difference ranging from 0.04 to 0.08 nm. An intermediate fluctuation difference is observed for the basic spine arginines of the K/N-LOX-1 monomers (the absolute value of their difference ranging from 0.02 to 0.04 nm) (Figure 7, panels C and D).

## Discussion

Discordant results have been reported on the association of the single nucleotide polymorphism c.501G>C of the *OLR1* gene to CAD/AMI. In one study, a higher frequency of LOX-1 gene variants in Japanese patients with MI than in controls was found [21]. While two different, but comparable studies reported a lower frequency of 501 SNP in patients with acute myocardial infarction than in controls, indicative of a positive association to MI [15], [20]. More recently, however, two studies failed to replicate an association of the coding c.501G>C SNP with CAD [19], [23]. In order to elucidate the mechanism by which the c.501G>C polymorphism may influence the risk of atherosclerosis and MI, we have performed a detailed biochemical and functional analysis of the p.K167N mutation of LOX-1 receptor.

We have analysed the effects of heterologous expression of p.K167N variant in mammalian fibroblasts and human endothelial cells. We show that both wt-LOX-1 and mut-LOX-1 variant display a stable dimeric structure and tend to form higher MW oligomers *in vivo*. When the two proteins are co-transfected, they form hetero-oligomers, in particular hetero-dimers and hetero-tetramers. Moreover, they have the same glycosylation/maturation process and the missense p.K167N mutation does not affect LOX-1 receptor trafficking to the plasma membrane. Importantly, when ectopically expressed, the amount of exposed receptors, at the steady state, is identical. However, at a functional level, the presence of p.K167N mutation in LOX-1 receptors reduces ox-LDL binding and uptake. In particular, the binding activity is 30–35% lower in case of homozygosity for N/N-LOX-1 expression and 20% reduced in case of heterozygosity, obtained by co-transfection of K/K167 and N/N167 DNA plasmids at a ratio 1∶1. It is worth noting that the presence of the C-terminal tags (myc- and V5-tags) in LOX-1 receptors and its variants does not have any influence on the reported effects, as demonstrated by transfection and functional analysis with the non-tagged proteins (18 and unpublished results).

ERK 1/2 phosphorylation is specifically stimulated by ox-LDL in LOX-1 expressing cells (12, 13 and Figure 4). Since p.K167N mutation affects ox-LDL binding activity, we next examined the activation of the extracellular signalling-regulated kinases ERK 1/2 in K/K167, K/N and N/N167 transfected cells. Expression of K/N and N/N167 LOX-1 receptors in fibroblasts resulted in a marked inhibition of ERK activation (85% reduction in case of homozigosis and 70% in case of heterozygosis) with respect to ERK activation in wt LOX-1 expressing cells, indicating the impairment of the ox-LDL-dependent intracellular response. ERKs belong to a family of mitogen-activated protein kinases associated to a variety of biological responses, depending upon the cell type, the stimulus and the duration of activation. These include also injury-induced tissue damage and apoptosis in some cell types and organs [24]. Although we do not have evidences on the downstream effects of the observed impairment of ox-LDL-dependent ERK phosphorylation, further studies are likely to clarify this important issue. It is worth noting that also expression of the mutant form of LOX-1 protein K267A LOX-1 blocks ERK1/2 activation [13]. Hetero-dimerization between mutated and wild-type LOX-1 receptors attenuate the expression of functional receptors and, therefore, the intake of ox-LDL inside the cells.

The G-to-C transition of LOX-1 gene results in the K-to-N change at 167, far from the basic spine arginines of the CTLD domain that represents the ligand binding domain (Figure 6D). From modeling studies, the first important effect of the single p.K167N mutation is an altered balance of charges in the network formed by E166, K167, E170 and K171. This network includes the mutation site and alterations, i.e. the uncoupled negative charge of E170, are observed either in the homozygous N/N-LOX-1 and in the heterozygous K/N-LOX-1 mutant. Secondly, it was observed an asymmetric flexibility of the basic spine residues, in particular in the N/N-LOX-1 mutant. We hypothesize that the variation of potential distribution, observed in N/N-LOX-1, is due both to an unbalanced network of charges and to an altered flexibility of the basic spine residues that, together, induce a marked inhibition of ox-LDL recognition. In the heterozygous K/N-LOX-1 mutant the unbalanced network of charges alters the potential distribution but is not supported by the basic spine arginines large asymmetric fluctuation, explaining the lower level of impairment.

Ox-LDL is considered to play a fundamental role in the entire process of atherogenesis. LOX-1 activation by ox-LDL binding rapidly induces a cascade of intracellular signalling leading to endothelial activation, cell proliferation, apoptosis and atherosclerosis [2]. Although LOX-1 is expressed at low level in normal conditions, its expression is highly activated by ox-LDL binding via intracellular signalling, indicating that pro-inflammatory conditions create a positive feedback that enhance endothelial dysfunction [9]. Interestingly, when *in vitro* differentiated human macrophages, derived from K/N167 heterozygous individuals were incubated with ox-LDL to study LOX-1 receptor synthesis and induction, we found a marked decrease in ox-LDL-dependent LOX-1 induction compared to the induction seen in differentiated human macrophages derived from K/K167 control individuals.

The single aminoacid p.K167N mutation of LOX-1 receptor induces decreased efficiency in ox-LDL binding and uptake, affects its signal transduction activity and, *in vivo*, results in a marked decrease in the induction of LOX-1 receptors upon stress stimuli in human macrophages. All these effects may have a marked influence on ox-LDL toxicity *in vivo*, and may affect the severity of CAD and atherosclerosis. Interestingly, the frequency of the heterozygous p.K167N LOX-1 is about 10% in Caucasian population, while homozygous for the N allele are very rare [15]. Unfortunately, whether the LOX-1 induction in human macrophages derived from the homozygous N/N167 individuals is even lower than in the heterozygosis could not be verified.

In conclusion, the lower LOX-1 activity in individuals carrying the p.K167N mutation reported in this paper suggests that the lysine residue at 167 is crucial for ligand binding activity of LOX-1 protein and for its function. This finding strengthens the idea that specific inhibition of LOX-1 receptor may be a valuable therapeutic strategy for combating atherosclerosis.

## Materials and Methods

### DNA constructs and mutagenesis

For the expression in mammalian cells, human LOX-1 was subcloned into pEF/myc-His and pEF/V5-His vectors (Invitrogen), LOXIN and R4 were subcloned into pEF/V5-His vector (Invitrogen) as previously described [18].

Generation of pscFvexpress-Sec-8H4 has been previously described [25]. Mutagenesis of Lys167 to Asn residue of Lox-1 was performed using QuikChange® Multi Site-Directed Mutagenesis kit purchased from Stratagene following the manufacture's protocol. Mutagenesis of pEF/V5-LOX-1 was carried out using the following primer 5′-CATTTAACTGGGAAAACAGCCAAGAGAAGTGC-3′. The mutation was confirmed by sequencing using ABI PRISM® 3130 XL Genetic Analyzer (Applied Biosystems).

### Antibodies and reagents

Mouse anti-myc IgG 9E10 (Invitrogen), mouse anti-V5 IgG (Invitrogen), polyclonal rabbit anti-myc (Santa-Cruz), polyclonal rabbit anti-ERK1/2 (Biosource, Invitrogen), mouse anti-phospho ERK1/2 (Cell Signaling Technologies), mouse anti-β-actin IgG (Affinity BioReagents) and rat anti-LOX-1 [18] were used as primary antibodies. Goat anti-rat IgG horseradish peroxidase (HRP) was purchased from Pierce, goat anti-mouse IgG HRP, donkey anti-rabbit IgG HRP and Rhodamine Red TM-X-conjugated AffiniPure donkey anti-mouse IgG from Jackson Immunoresearch. Peptide-N-glycosidase F (PNGase-F) was purchased from New England Biolabs (NEB), and proteases inhibitor cocktail set III from Calbiochem.

### Cell cultures and transfection

COS cells were grown in DMEM medium (Euroclone) supplemented with 10% foetal bovine serum (Gibco) and 100 U/ml penicillin/streptomycin (Euroclone). Human monocytes were isolated from peripheral blood mononuclear cells (PBMCs) from buffy coats of volunteers and promoted their transition to macrophages *in vitro* as described [26]. COS cells were transiently transfected with Superfect (Qiagen) following the manufacturer's instructions, with a DNA/ transfectant reagent ratio (w/v) of 1∶5.

### Western blot and Immunoprecipitation

Transfected COS cells and human primary macrophages were washed twice with ice-cold phosphate buffer saline, lysed for 20 minutes at 4°C in ice-cold extraction buffer (EB) containing 10 mM Tris/HCl pH 7.6, 100 mM NaCl, 10 mM EDTA, 0.5% Nonidet P40, 0.5% sodium deoxycholate, proteases inhibitor cocktail set III (0.1 mM AEBSF hydrochloride, 0.5 µM aprotinin, 5 mM Bestatin, 1.5 µM E-64, 10 µM Leupeptin, 1 mM Pepstatin A and 1 mM phenylmethylsulfonyl fluoride) and centrifuged for 15 minutes at 4°C at 15,000×g. The supernatant fraction was analysed by SDS-PAGE in 12% acrylamide gels and transferred to polyvinylidene difluoride (PVDF) membranes (Amersham Bioscences) for 16 h at 30 V. Immunoreactive bands were visualized by enhanced chemiluminescence (ECL, Sigma). For separation of oligomeric forms, proteins were separated by NuPAGE 3–8% Tris-Acetate Gels (Invitrogen) in non reducing conditions.

Immunoprecipitation with Mab anti-V5 and rabbit anti-myc IgG was performed as previously described [18]. For removal of N-linked glycans, clarified cellular lysates were MeOH-precipitated and digested with PNGase-F as previously described [18].

### Immunofluorescence analysis and surface labelling quantification

Cell membrane immunofluorescence was carried out as described [25] using Mab anti-V5 as primary antibody and Rhodamine RedTM-X-conjugated AffiniPure donkey anti-mouse IgG as secondary antibody. Samples were examined with a DMRA Leica fluorescence microscope, equipped with CCD camera and with a confocal microscope (Nikon Instruments Spa, C1 on Eclipse TE200; EZC1 software). CytoELISA assay for quantification of membrane expressed proteins was performed as previously described [18].

### ERK 1/2 activation

COS cells were transiently transfected with K/K167, K/N167 and N/N167 LOX-1-V5 and LOXIN-V5 as described above. 48-h after transfection, non-transfected and transfected COS cells were incubated with different concentration (from 10 to 100 µg/ml) of ox-LDL for 10 minutes at 37°C. After treatment, cells were harvested, lysed and cell extracts were separated with SDS-PAGE in 12% acrylamide gels and blotted. The levels of ERK1/2 were detected by polyclonal antibodies directed against the C-terminal region of the human protein and the activated kinase was detected with mouse monoclonal antibodies directed against the phospho-peptide corresponding to residues surrounding Thr202/Tyr204. Immunoreactive bands were visualized by ECL. Densitometric measurements were performed on a VersaDoc Imaging System (BioRad).

### Ox-LDL preparation, labelling and fluorometric assay

Human LDL was prepared from fresh healthy normolipidemic plasma of volunteers by ultracentrifugation [27]. LDL was oxidised and labelled with 1,1′-dioctadecyl-3,3,3′,3′-tetramethyllindocarbocyanine perchlorate (DiI) as previously described [18]. 24 hours after transfection cells were incubated with (DiI)-labelled ox-LDL in serum-free medium on ice for 1 hour in binding assay and at 37°C for 4 h in uptake assay. DiI fluorescence was observed with a DMRA Leica fluorescence microscope, equipped with CCD camera. Quantitation of ox-LDL receptor activity in cells was assayed by DiI extraction in isopropanol [28] and fluorescence determined in a Perkin Elmer spectrofluorometer with excitation and emission wavelengths set at 520 and 578 nm, respectively.

### Molecular dynamics simulation

LOX-1 protein coordinates were obtained by X-ray [4], [5] as stored in the Protein Data Bank (PDB) (www.rcsb.org/pdb) selecting the 1YPQ file, having the highest resolution (1.4 Å) [4]. In this structure, due to the absence of the LOX-1 neck domain, the terminal ends of the CTLD domain are not uniformly determined by X-ray diffraction. To avoid the presence of asymmetric N and C termini the subunits sequences have been made of the same length. Following the sequence numbering given in the 1YPQ PDB file, 4 residues (from Arg136 to Asn139) have been removed from the N-terminus of monomer B because they are not present in the monomer A, and 3 residues (from Arg271 to Gln273) in the conformation detected in the 1YPO LOX-1 structure [4], have been added to the C-terminus of both monomers. The dioxane molecule, bound within the largest tunnel chamber, has been removed from the structure whilst the 388 water molecules have been maintained and mixed with those of the built simulation boxes. The modelling has been carried out using the SYBYL 6.0 program (Tripos Inc. 1699, South Hanley Road St. Louis, Missouri, 63144, USA).

The system topologies have been obtained with the AMBER LeaP module [29], and modelled with the all-atoms AMBER95 force field [30], [31]. The proteins have been immersed in rectangular boxes filled with TIP3 water molecules [32] and the three systems have been neutralized adding the necessary amount of counterions in electrostatically preferred positions. The two systems have been simulated in periodic boundary conditions, using a cut-off radius of 9.0 Å for the non-bonded interactions, and updating the neighbour pair list every 10 steps. The electrostatic interactions have been calculated with the Particle Mesh Ewald method [33], [34]. The SHAKE algorithm [35] has been used to constrain all bond lengths involving hydrogen atoms. The systems were simulated for a total of 15 ns at constant temperature of 300 K using Berendsen's method [36] and at a constant pressure of 1 bar with a 2.0 fs time step. Pressure and temperature coupling constants were 0.4 ps. The atomic positions were saved every 250 steps (0.5 ps) and the last 10 ns has been used for the analysis. The systems were simulated at CASPUR research center of Rome, Italy (Inter Universities Consortium for Supercomputing Applications) on Power 4 IBM parallel computers by using an 8 CPU cluster. The RMSF was calculated over the equilibrated MD trajectories removing the global translations and rotations. The time evolution of RMSD has been monitored using the GROMACS MD package version 3.1.4 [37]. The salt bridges have been calculated using the VMD program version 1.8.5 [38].

### Electrostatic isopotential surfaces

The electrostatic isopotential surfaces of K/K167, N/N167 and K/N167 LOX-1 receptors were calculated for selected frames of the MD trajectories solving the Poisson-Boltzmann equation using the DelPhi algorithm [39], as implemented in the GRASP program version 1.2 [40]. The program numerically solves the Poisson-Boltzmann equation by finite difference method (FDPB) [41] and visualises the electrostatic isosurfaces on the rendered molecular surface. Net charges were assigned to all ionizable groups considered in their standard protonation state at pH 7 [42].

### Statistical data analysis

Data are reported as means±S.E. Differences were tested for significance using one-way ANOVA followed by Bonferoni's test. The significance level was chosen as p>0.05.

## Supporting Information

Figure S1 (0.49 MB TIF)
