# Supplementary figures and images for: Functional Analysis and Molecular Dynamics Simulation of LOX-1 K167N Polymorphism Reveal Alteration of Receptor Activity
Source: PLoS One. 2009 Feb 27;4(2):e4648. doi: 10.1371/journal.pone.0004648 (PMC2645694; doi:10.1371/journal.pone.0004648)

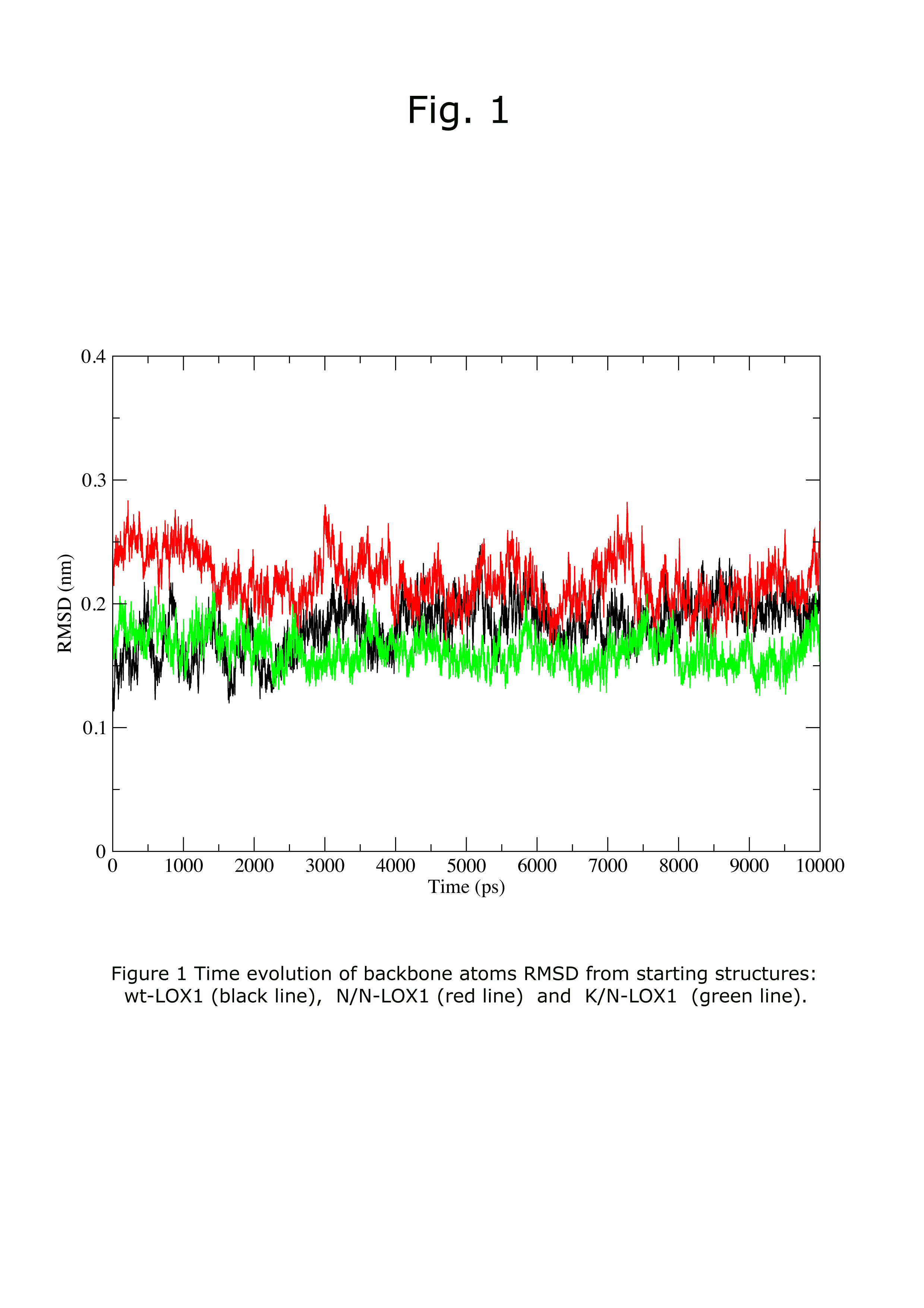

Supplement: Figure S1 — (0.49 MB TIF) [file pone.0004648.s001.tif]
